# Research on an Intelligent Mining Complete System of a Fully Mechanized Mining Face in Thin Coal Seam

**DOI:** 10.3390/s23229034

**Published:** 2023-11-08

**Authors:** Bo Ren, Ke Ding, Lianguo Wang, Shuai Wang, Chongyang Jiang, Jiaxing Guo

**Affiliations:** 1 State Key Laboratory of Mining Response and Disaster Prevention and Control in Deep Coal Mines, Anhui University of Science and Technology, Huainan 232001, China; 02140873@cumt.edu.cn (B.R.); swang@cumt.edu.cn (S.W.); 2 State Key Laboratory for Geomechanics and Deep Underground Engineering, China University of Mining and Technology, Xuzhou 221116, China; cumt_lgwang@163.com (L.W.); jiangcy32942@163.com (C.J.); guo1jiaxing@126.com (J.G.)

**Keywords:** intelligent mining, control system, equipment selection, reduce people and improve efficiency, collaborative control, complete sets of equipment and technology, thin coal seam, fully mechanized coal mining face

## Abstract

The mining environment of thin coal seam working faces is generally harsh, the labor intensity is high, and the production efficiency is low. Previous studies have shown that thin coal seam mining finds it difficult to follow machines, does not have complete sets of equipment, has a low degree of automation, and has difficult system co-control, which easily causes production safety accidents. In order to effectively solve the problems existing in thin coal seam mining, Binhu Coal Mine has established intelligent fully mechanized mining and actively explored automatic coal cutting, automatic support following, and intelligent control. The combination of an SAC electro-hydraulic control system and SAP pumping station control system has been applied in 16,108 intelligent fully mechanized coal mining faces, which realizes the automatic following of underground support and the control of adjacent support, partition support, and group operation; the automatic coal cutting of the shearer is realized by editing the automatic coal-cutting state of the shearer and adjusting the automatic parameters. A centralized control center is set up, which realizes the remote control and one-button start–stop of working face equipment. Through a comparative analysis of 16,108 intelligent fully mechanized mining faces and traditional fully mechanized mining faces, it is found that intelligent fully mechanized mining faces have obvious advantages in terms of equipment maintenance, equipment operation mode, and working face efficiency, which improve the equipment and technical mining level of thin coal seam. The application of intelligent mining in Binhu Coal Mine has a great and far-reaching impact on the development of thin coal seam mining technology in China.

## 1. Introduction

The coal resources in thin coal seam are widely distributed, however, its output is far lower than the proportion of reserves, resulting in an imbalance between outputs and reserves, and some resources have not been mined for a long time. In order to improve the utilization rate of resources, ensure the balance of mine production capacity, and realize the sustainable development of mines, it is necessary to mine thinner coal seam reserves and implement “thin–thick” and “fat–thin” matching mining [1,2,3].

With the continuous exploitation of coal resources in eastern China, the proportion of coal resources in western China is rising. By 2020, the proven recoverable reserves of thin coal seams were about 600 million tons, accounting for about 20% of the total coal reserves in China, and most of the thin coal seam resources are concentrated in the central and western regions of China, such as Sichuan Province, whose thin coal seam resources account for 51.8% of the coal resources in this province; Shanxi Province is one of the provinces with the richest thin coal seam resources in China [4]. Thin coal seam resources account for 17.6% of the province’s coal resources, and the mining reserves of thin coal seam are 1.38 billion tons, mainly distributed in Datong, Ningwu, Hedong, Xishan, Luliang, Wutai, and Xinzhou; the thin coal seam resources in Shandong Province are mainly distributed in Xinwen, Longkou, Yanzhou, Zaozhuang, and Jining [5,6]. Thin coal seam resources account for 16.8% of the coal resources in Hebei Province, mainly distributed in Xingtai, Handan, Zhangjiakou, Chengde, and other areas; the thin coal seam resources in Anhui Province are mainly distributed in Huaibei, Huainan, Suzhou, and Fuyang [7,8].

There are many problems and inconveniences in thin coal seam mining, such as a bad working face environment and low and narrow working face space. As a result, people cannot walk upright, labor intensity is high, there are many gangue faults, and some working faces cannot even enter. Moreover, it is difficult to follow machines and repair the equipment, which easily causes production safety accidents [9,10,11]. Therefore, the automation and intelligence of equipment and technology are the key and inevitable choice to realize the safe and efficient mining of thin coal seam, and it is also the development direction of modern mine construction [12,13]. According to the actual situation of thin coal seam mining in the Western Donbas mines, a new progressive method is put forward, which can effectively reduce the output of gangue, reduce the pollution to the environment, and bring about remarkable economic benefits [14]. In addition, the coal industry is gradually developing from manual coal mining and semi-mechanized coal mining to mechanized, comprehensive mechanized, and automatic coal mining, and has begun to move forward from automatic mining to intelligent mining [15,16,17]. With major breakthroughs in new generation information technologies such as 5G communication, big data, and Internet of Things, the upgrading and transformation of the coal mining mode from automation to intelligence has received technical support. The concepts of smart coal mines and intelligent mines are gradually becoming clear, and the standards and specifications are gradually being completed, which lays a foundation for theor large-scale popularization and application [18,19,20,21].

Currently, domestic and international experts and scholars have extensively explored and researched intelligent coal mining, yielding abundant results. Wang [22] reported an intelligent system for mechanized mining equipment with Chinese independent intellectual property rights. An intelligent mining model was established to realize unmanned operation and single person inspection at the working face. Basarir [23] and Massinaei [24] achieved the intelligent, fully mechanized mining of super-thick coal seams. This was accomplished using hydraulic caving support for high-top coal in large-scale mines, an intelligent coal tunnel system, and crucial intelligent control technology, thus providing reference for the intelligence-led construction of deep, thick coal seam. Tian [25] put forward five key research and development directions of intelligent coal mines, analyzing the key technical problems involved in each research and development direction. They pointed out that intelligent coal mines are large-scale systems, and they should follow the theory of system engineering and mining law to develop the integrated management, development, and operation platform of an intelligent coal mine giant system. Shen [26] summarized the four stages of intelligent mining, analyzed the production traits and technical prerequisites at different stages, proposed a control theory model within the intelligent adaptive mining technology mode, and advanced towards intelligent mining using essential new technologies. Yang [27], in order to solve the problem of large mining height comprehensive mining faces with many workers and a high labor intensity, analyzed the technical difficulties such as the precise control of rib spalling, soft underframe and equipment reliability, perception accuracy, poor coordination, and other technical difficulties in the intelligent comprehensive mining of large mining height working faces. The visualization remote intervention technology route was adopted to realize the intelligent normal mining of a large mining height comprehensive mining working face, which achieves the purpose of reducing personnel and improving efficiency. The article proposed using an energy-based principle of ground control in a fully mechanized longwall, which means the adjustment of the volume of power fluid by-pass from the head ends of the hydraulic props of the powered support units to the pressure line of the hydraulic system of the fully mechanized longwall mining system during the subsidence of roof rocks [28]. Wu [29] studied the current situation and development trend of intelligent mining technology in a comprehensive mining face, and divided the intelligent mining technology in the comprehensive mining face into two stages, namely, intelligent unmanned mining in the visualization remote intervention mode and intelligent unmanned mining in the adaptive mode. He believes that mine large section roadway deformation intelligent control technology, mine tunneling machine intelligent technology, anchor bracket intelligent technology, transportation system intelligent technology, and video monitoring intelligent technology will be the key technologies of comprehensive mining face intelligentization. Reflecting on the current state of visual remote intervention, robot-assisted inspection, and inertial navigation technologies, Huang [30] introduced four-dimensional intelligent coal mining system infrastructure which encompasses “perception, decision-making, execution, and operation and maintenance”. Furthermore, they outlined the technological advancements necessary to progress key intelligent coal technologies. This plays an important role in building an intelligent mining system. Similarly, Zhan [31], in order to improve the degree of automation and intelligence of longwall mining, in response to the problems of unknown information of the mining area and the insufficient objectivity of decision making in the production process during the construction process of some intelligent coal mining enterprises, proposed a four-aspect global model for the construction of a transparent longwall mining face on the basis of analyzing the special requirements of automated mining in coal mining enterprises. The structure of the whole transparent longwall mining face was designed from the four dimensions of intelligent application, informatization, transportation, and sensing. This has good reference significance for the system design of intelligent working faces in thin coal seam. Si [32], by studying the intelligent production mode and status quo of intelligent technical equipment, presented the subsequent focus areas for intelligent technical equipment in fully mechanized mining faces. These research efforts primarily targeted the intelligent mining of substantial coal seam and top-coal caving faces. However, when compared to vast coal seams, the mining conditions of thin coal seams are more complex due to the lower thick bottom of the coal seam and significant variance in angles, folds, and faults. Bołoz [33] introduced, in detail, the common mining methods in thin coal seam underground mining and the disorders in the mining machines used in the methods of Highwall, Auger, and Punch longwall mining, along with their types and variations. The article proposed a constructive technical solution to increase the contact adaptivity of the mechanical roof support. The developed solution of the mechanized support section is able to adapt to changing mining and geological conditions in the process of the excavation of the mining pillar, which increases the efficiency and safety of coal excavation in the longwall [34]. Additionally, the intelligent development process faces numerous issues such as ambiguous concepts and technical connotations and indistinct mining modes and technical routes, among others.

Analyzing the above, intelligent mining is a hot issue. This paper synthesizes the advantages of domestic and foreign thin coal seam mining technology, aiming at the intelligent mining of fully mechanized coal mining faces in thin coal seam, actively explores automatic coal cutting, automatic support following machines, and intelligent control, and realizes the linkage control of coal cutting, traction, transportation, and support moving. Finally, the production automation, intelligent coal cutting, information management, and unmanned working faces of thin coal seam mining are realized.

## 2. General Situation of Intelligent Fully Mechanized Coal Mining Face in Thin Coal Seam

### 2.1. Geological Conditions

**Figure 1:**
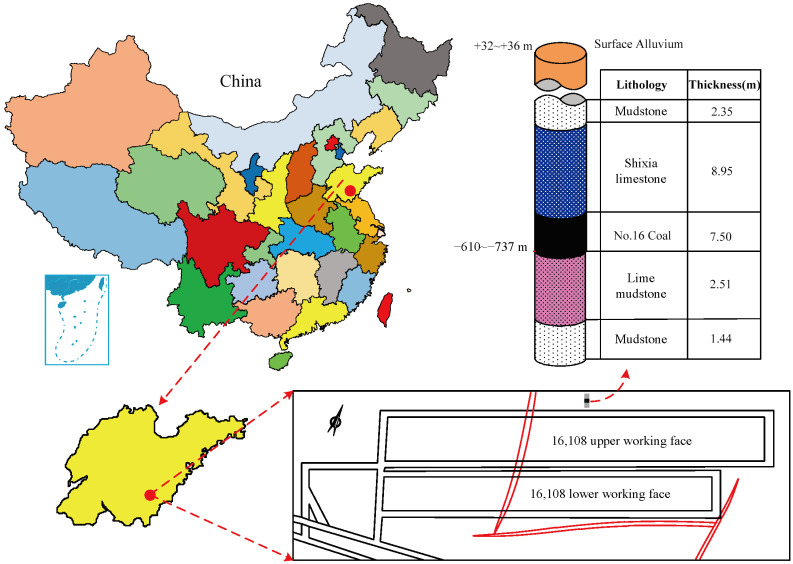
Location map of working faces.

The 16,108 intelligent fully mechanized coal mining faces in Binhu Coal Mine are located in 161 mining areas, mainly mining 16 coal seams of the Taiyuan Formation of Upper Carboniferous. The ground elevation of the working face is +32~+36 m, the working face elevation is −610. 0~−737.0 m, the average buried depth is 707.5 m, the coal seam thickness is 1.10~1.45 m, and the average thickness is 1.29 m. The strike length of the working face is 1400 m and the inclined length is 220 m, as shown in Figure 1. The occurrence state of the coal seam is relatively stable, the strike of coal seam is 167~199, the dip is 257~289, and the occurrence is gentle. The direct roof of the working face is ten lower limestone, with vertical fissures developed, the roof condition is hard, and the floor is sandy mudstone, which is semi-hard and relatively complete. The main structure of 16,108 intelligent fully mechanized coal mining faces in Binhu Coal Mine belongs to a monoclinic structure, and the average dip angle of the coal seam is 11. No fold development is found from the exposure of two roadways, and only one fault is exposed, with a drop of about 1.5~5.5 m.

### 2.2. Intelligent Mining Equipment Matching of Fully Mechanized Coal Mining Face in Thin Coal Seam

The intelligent and fully mechanized mining face of the Binhu Coal Mine 16108 is equipped with the MG400/870-WD double drum shearer produced by Shandong Zhongmei Intelligent Equipment Co., Ltd., the SGZ764/500 scraper conveyor produced by Shandong Xingye Machinery Accessories Co., Ltd., and the SZZ730/160 bridge conveyor produced by Luoyang Yuanjian Mining Equipment Co., Ltd. A total of 100 supports were selected for the intelligent and fully mechanized mining face of 16108, with 96 units of ZY400/09/18D electro-hydraulic control hydraulic supports as the main supports, and 4 units of ZT400/12/24YD electro-hydraulic end supports, all produced by Jining Huaming Mining Machinery Equipment Co., Ltd.

The reliability of equipment should be considered in the equipment matching of intelligent fully mechanized coal mining faces in thin coal seam. First, whether it can meet the requirements of an intelligent fully mechanized coal mining face for the production of 1.1 million t/a; the second is to ensure that the selection of various equipment should be reasonable, ensure the reliability and stability of the coal flow system, and ensure the rapid advancement of the working face; and third, whether the equipment of the working face and transportation lane can realize coordinated and centralized control. The equipment models of the 16,108 intelligent fully mechanized coal mining faces and transportation roadways are shown in Table 1.

## 3. Intelligent Mining System of Fully Mechanized Coal Mining Face in Thin Coal Seam

**Figure 2:**
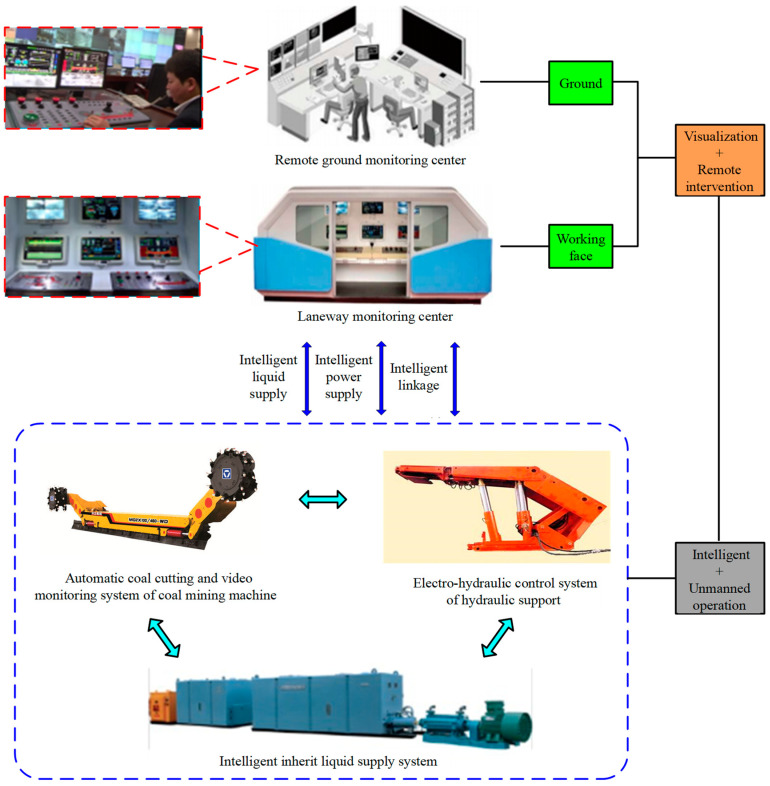
Composition of intelligent mining system for fully mechanized coal mining face in thin coal seam.

The intelligent mining system of a fully mechanized coal mining face in a thin coal seam of 16,108 working faces includes an electro-hydraulic control system of hydraulic support, automatic coal cutting and video monitoring system of shearer, intelligent integrated liquid supply system, and remote centralized control system. A total of 16,108 intelligent fully mechanized coal mining faces realize automatic support moving through the electro-hydraulic control system, and display and monitor the position of shearer and support in real time through sensors installed on the shearer and support, so as to ensure that the support automatically moves with the shearer, realizing the automatic coal caving of the support by using the time parameters and stroke height of the swing beam. Through the automatic coal cutting of the shearer and video monitoring system, the coal cutting state of the shearer is edited and the position and direction of the shearer (upward or downward) are monitored to realize the automatic coal cutting of the shearer according to coal mining technology. The intelligent linkage of multi-pumping stations and remote multi-machine control are realized through intelligent integrated liquid supply and a remote centralized control system. In a word, the working face adopts a variety of sensors, and realizes the realization of equipment in the whole working face through program control. The coordinated management and centralized control of equipment in the comprehensive mechanized coal mining face make the equipment run continuously, efficiently, and safely. The composition of the intelligent mining system in a fully mechanized coal mining face in thin coal seam is shown in Figure 2.

### 3.1. Electro-Hydraulic Control System of Hydraulic Support

**Figure 3:**
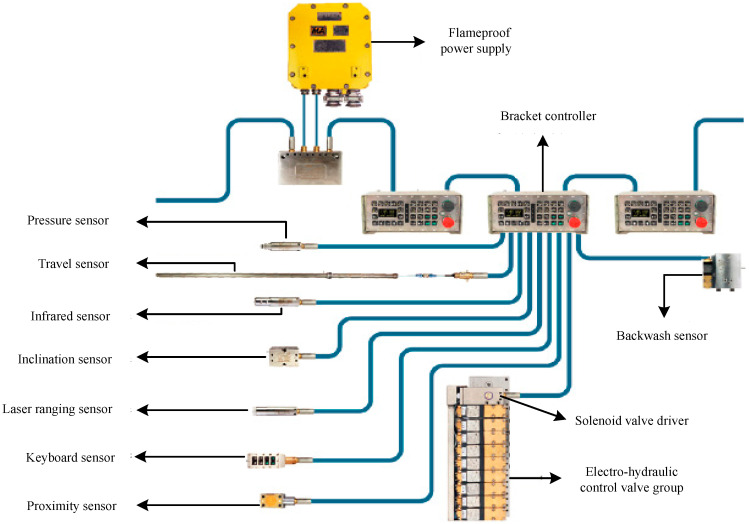
Structure Schematic Diagram of SAC Electro-hydraulic Control System for Hydraulic Support.

The electro-hydraulic control system of hydraulic support adopts an SAC electro-hydraulic control system, which is mainly composed of three aspects, as shown in Figure 3.

(1) All basic frames of the 16,108 intelligent fully mechanized coal mining faces are equipped with one controller, eight sensors, one driver, and one electro-hydraulic control valve group, which, together, constitute the most basic control unit. The controller is the core of the control unit. It is a microcomputer which is equipped with operating software and a human–computer interaction interface. It is a platform for employees to operate. The detection link includes five types and eight kinds of sensors, which are front and rear column pressure sensors, infrared receivers, push stroke sensors, guard proximity sensors, inclination sensors (main top beam, shield beam, and tail beam), and rear swing beam stroke sensors. The executive link includes a driver and an electro-hydraulic valve group, which drives the retracting and extending actions of various supports.

(2) All support controllers in the 16,108 intelligent fully mechanized coal mining faces are interconnected to form a communication network system, and data communication among controllers is realized by bus technology. After the controllers are interconnected, the main functions such as adjacent/separated support control, remote control, single support or group automatic control, emergency stop of the whole line, state and fault information display, and so on are realized. The SAC system is equipped with the necessary hardware (connector and isolation coupler, etc.) and corresponding software for data communication and control function for controller interconnection. A special controller, called a signal converter, is installed and connected in the No. 2 support of 16,108 intelligent fully mechanized coal mining faces, which can provide more abundant and perfect services for the controller system of the working face.

(3) The underground main control computer is set up in the air inlet lane of the 16,108 intelligent fully mechanized coal mining faces, and connected with the support controller of the working face through the network through the signal converter to become the upper-level control machine. The main control computer runs its own software, collects and stores the data and parameters collected and transmitted from the support controller of the working face, calls and displays these data parameters at any time, and monitors the working conditions and action states of the support.

The SAC electro-hydraulic control system of the hydraulic support can complete various action functions of the support, and the support can realize automatic control in groups, including automatic support moving, automatic push-slip, automatic pull-back slip, automatic extension and retraction of the protective plate, automatic extension and retraction of the telescopic beam, automatic spraying, and so on. The main purpose of the SAC electro-hydraulic control system of the hydraulic support is to realize the function of automatically following the machine and moving the frame with the shearer. When the shearer runs from the tail to the head to clear the floating coal to the No. 80 support, it triggers the push-slip of the No. 43~50 supports, which forms a serpentine oblique cutting feed section, and the No. 1~42 supports carry out full-stroke push-slip, from the No. 1~42 supports in sequence. When the shearer runs from the head to the tail to the No. 20 support, it triggers the push and storage action of the No. 51~No. 100 supports. After the shearer, the No. 6 support lagging behind the shearer begins to pull the support, which is completed in turn.

### 3.2. Automatic Coal Cutting and Video Monitoring System of Shearer

**Figure 4:**
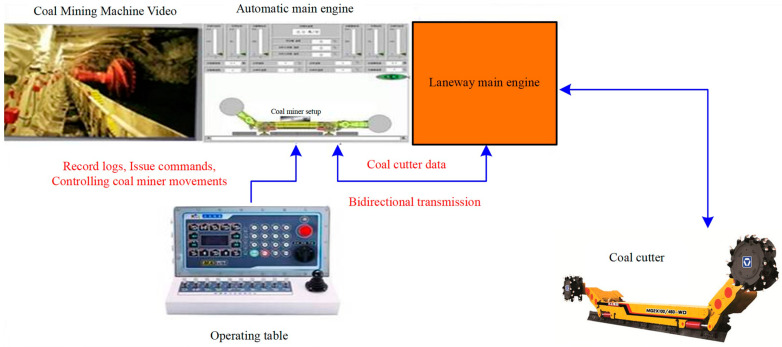
Schematic diagram of automatic coal cutting and video monitoring system of shearer.

The automatic coal cutting of shearer and the operation of the video monitoring system in 16,108 intelligent fully mechanized coal mining faces are shown in Figure 4. The automatic coal cutting system of the shearer adopts a memory cutting TDECS system which is suitable for the coal mining technology of working faces. Firstly, the automatic coal cutting state table is edited, and 22 states are edited altogether. The setting process is: automation → status table → editing status; Automation → Basic Settings → State Based, traction automation and lateral inclination compensation are set to “start”; Parameters → driver settings → starting the motor “left wireless controller”; the F1 + Auto key can enable the left remote controller to control the automatic coal cutting of the shearer, and the shearer driver can properly intervene and fine-tune the coal cutting state of the shearer, including the coal cutting height and coal cutting speed.

At the same time, in order to ensure real-time dynamic grasp of the working face, one camera is installed on every three hydraulic supports in the working face, which is installed on the front main top beam of the front column. There are 34 cameras installed in the working face, 17 of which face the working face and 17 face the coal wall. These 34 cameras can be automatically switched according to the position of the shearer to ensure that the coal cutting environment of the shearer is always under monitoring. In addition, the 16,108 intelligent fully mechanized coal mining faces are also equipped with cameras in special areas, including the front slipper, rear slipper, transfer machine, front beam detection of the No. 2 support, and train centralized control center, etc. These special area cameras can dynamically monitor the operation and coal flow of these key parts in real time. Three video monitors are installed in the centralized train control center and the ground dispatching sub-control center of the transportation lane of the 16,108 intelligent fully mechanized coal mining faces, 1 of which displays the video of special area fixedly, 1 of which displays the video of working face facing coal wall, and 1 of which displays the video of working face sliding forward.

### 3.3. Intelligent Integrated Liquid Supply System

The intelligent integrated liquid supply system adopts an SAP intelligent integrated liquid supply system, which consists of four parts: a pumping station, liquid tank, control system, and multi-stage filtration system. It is mainly automatic equipment integrating a pumping station, electromagnetic unloading automatic control, PLC intelligent control, frequency conversion control, multi-stage filtration, emulsion automatic proportioning, system running state recording, and uploading, and is a complete liquid supply system for fully mechanized coal mining faces. According to the actual needs of the working face, a liquid supply system with different flow levels and different configuration requirements can be designed, and it is compatible with most pumping stations, combined switches, and frequency converters.

The system is equipped with a multi-stage filtration system, including an inlet water filtration station, water addition filter, high-pressure filtration station, and return liquid filtration station. Through the combination of filter elements with different precisions and flows, the cleaning of the hydraulic medium and the stability of the system are ensured. At the same time, the automatic emulsion proportioning device is adopted in the working face to realize stable automatic emulsion proportioning. In addition, the emulsion pump station adopts electromagnetic unloading control to realize the no-load start and stop of the emulsion pump, and has a variety of intelligent control modes such as single control, upper control, and joint control, which realize the intelligent linkage of multiple pump stations and can realize the intelligent start and stop control of “secondary and standby” pumps according to the liquid consumption situation of the working face. The intelligent control system can automatically detect, display, and control the whole system in real time.

### 3.4. Remote Centralized Control System

**Figure 5:**
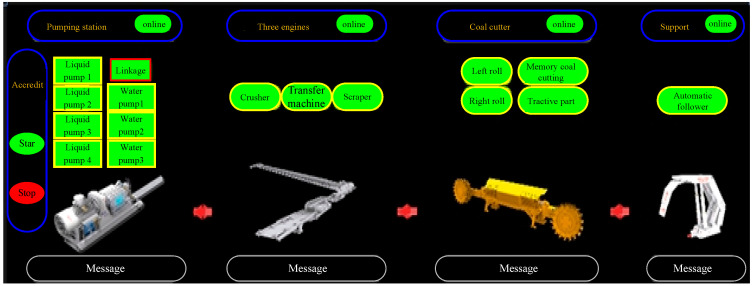
One-button start–stop host screen.

Ethernet and intelligent remote control systems are integrated into the 16,108 intelligent fully mechanized coal mining faces of Binhu Coal Mine to realize the remote centralized control of a ground and transportation lane monitoring center. In the centralized control center, various data of the shearer, hydraulic support, scraper conveyor, transfer machine, crusher, emulsion pump station, spray pump station, and other equipment can be displayed and monitored in real time, and the remote start–stop control of working face equipment can be realized at the same time. A one-button start–stop host screen is shown in Figure 5.

The successful application of a remote centralized control system realizes the remote group cooperative control of complete sets of equipment in fully mechanized coal mining faces, and provides the real-time production and safety information of underground coal mining faces for ground production and management personnel. The remote centralized control system has powerful functions, simple operation, and reliable operation, which effectively improves the production management efficiency of fully mechanized coal mining faces, improves the safety level of the working face, reduces the labor intensity of workers, improves the production efficiency, and is a favorable guarantee for a high yield and high efficiency of the working face.

## 4. Application Effect of Intelligent Mining in Fully Mechanized Coal Mining Face of Thin Coal Seam

The mining of 16,108 intelligent fully mechanized coal mining faces in Binhu Coal Mine has realized the intelligent cooperative control of complete sets of equipment in fully mechanized coal mining faces. It includes key technologies such as automatic coal cutting by a shearer (automatic coal cutting by a shearer includes oblique cutting feed, triangular coal cutting, bottom coal sweeping, and the upper or lower tail of the whole cutter, etc.), automatic support following (including following machine moving support, pushing and sliding, pulling and sliding, and automatic fluid replenishment of support, etc.), and remote centralized control of working face equipment. Compared with ordinary working faces, 16,108 intelligent fully mechanized working faces have obvious advantages in terms of equipment maintenance, equipment operation mode, and working face efficiency.

### 4.1. Maintenance of Intelligent Mining Equipment

**Figure 6:**
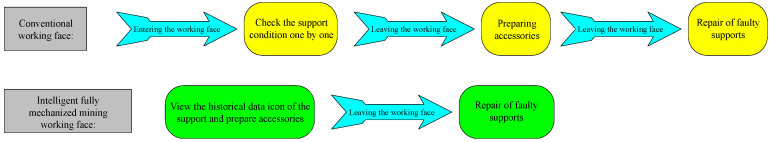
Equipment Maintenance Mode Control Chart.

The equipment maintenance mode of a traditional working face and intelligent fully mechanized working face is shown in Figure 6. The traditional equipment maintenance and repair requires the repairman to go into the working face to check each support one by one, record the support found to have problems, and prepare accessories to enter again for maintenance after leaving the working face as well. Moreover, through the comparison in Figure 6, it can be seen that the equipment maintenance under the intelligent mining system only needs the repairman to know the faulty support on the ground, which eliminates the process of entering the working face to check the support one by one, greatly shortens the labor time, improves the work efficiency, and ensures the safety of employees. At the same time, in the mining process of 16,108 intelligent fully mechanized coal mining faces, a clear water filtering device and softened water device are added, and automatic proportioning and emulsion filtering devices are added at the same time. The whole working face requires a high water quality, so it plays a very good role in protecting equipment maintenance.

### 4.2. Operation Mode of Intelligent Mining Equipment

**Figure 7:**
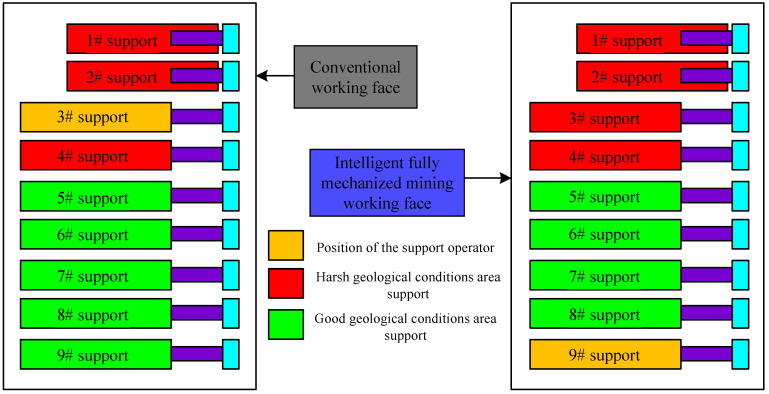
Control diagram of bracket operation mode.

The operation mode of traditional working face equipment is that traditional support control adopts temporary support operation, and the shearer uses a remote controller to follow the machine. People move back and forth in the working face and squat repeatedly, which is not only labor intensive and takes a long time, but also occupies more people and has a low efficiency. The equipment operation mode of an intelligent fully mechanized coal mining face is to use the intelligent mining system of a fully mechanized coal mining face in thin coal seam to organize production according to the principle of “unmanned equipment operation and patrolled working face”, that is to say, the working face is equipped with shearer memory cutting and support automatic following (including automatic following and moving support and automatic group pushing and sliding), and the working face personnel correct the engineering quality. It fundamentally avoids the disadvantages of traditional equipment, greatly improves efficiency, and reduces staffing. At the same time, for the support, in the face of harsh geological conditions, as shown in Figure 7, the operators of the traditional working face need to work in the cracks of dangerous support, and the safety of employees cannot be guaranteed, while the operators of the intelligent fully mechanized working face only need to complete their work outside the dangerous support area, which greatly improves the safety of employees.

### 4.3. Efficiency of Intelligent Fully Mechanized Coal Mining Face

**Figure 8:**
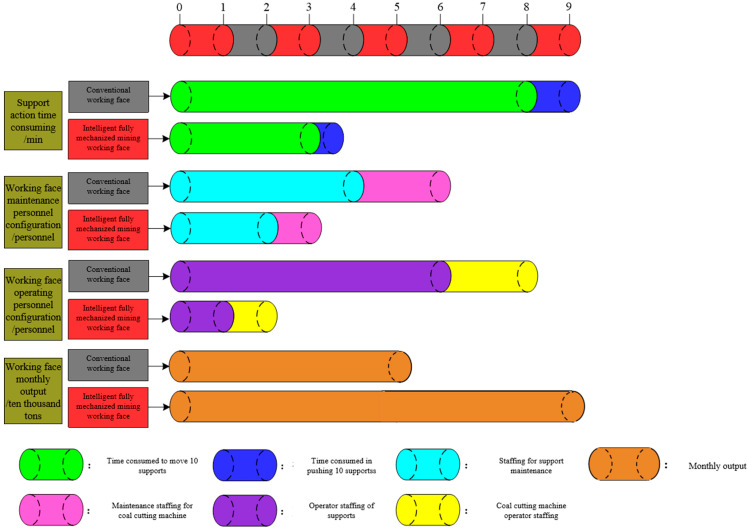
Comparison of working face efficiency.

The efficiency comparison between traditional working faces and intelligent fully mechanized working faces is shown in Figure 8. It takes 8 min for the traditional working face bracket to move 10 brackets and 1 min to push and slip 10 brackets; however, it takes only 3 min to move 10 supports in an intelligent fully mechanized coal mining face, while it takes only 10 s to push and slip, and the efficiency is 2.8 times that of traditional supports. The time for moving the frame at one time is reduced from 90 min to 32 min. Compared to the traditional working face, the time for moving the frame at one time is reduced by 64.8%. The number of equipment maintenance personnel in the working face is reduced from six to three in the traditional working face, and the number of equipment operators in the working face is reduced from eight to two in the traditional working face, with remarkable results in reducing personnel and improving efficiency. Compared to the traditional working face, the number of underground workers in the intelligent fully mechanized working face is only five, which is 35.7% that of the traditional working face, greatly reducing the number of underground workers. The monthly output of 16,108 intelligent fully mechanized coal mining faces is 90,000 tons, which is 1.8 times that of traditional coal mining faces. By comparing the adjacent traditional working faces of the mine, there are four cycles every day with a footage of 0.7 m, and the 16,108 intelligent fully mechanized working faces increases to seven cycles every day with a footage of 0.7 m. The shutdown time for moving racks is reduced from 360 min to 224 min per day, the net machine time for coal mining is increased from 480 min to 840 min, and the advancing speed of the working face increases by more than 75%.

## 5. Conclusions and Recommendations

(1) Based on the actual situation of 16,108 working faces in Binhu Coal Mine, the intelligent mining support equipment of a fully mechanized working face in thin coal seam is determined, which is mainly composed of a shearer, scraper conveyor, bridge conveyor, and electro-hydraulic control hydraulic support.

(2) On the basis of the reasonable selection of supporting equipment, an intelligent mining system for a fully mechanized coal mining face in thin coal seam is formed, including an electro-hydraulic control system of hydraulic support, automatic coal cutting, and the video monitoring system of a shearer, intelligent integrated liquid supply system, and remote centralized control system. The automatic coal cutting of the shearer, automatic follow-up of the support, and the remote centralized control of the working face equipment are realized. The successful mining of this working face proves the feasibility of the remote group cooperative control technology of complete sets of equipment in fully mechanized coal mining faces in thin coal seam, and accumulates experience for the intelligent mining of fully mechanized coal mining faces in thin coal seam.

(3) Compared to the traditional working face, the number of people required underground in the intelligent fully mechanized working face is only five, which is 35.7% of the traditional working face; compared to the traditional working face, the time for single frame moving is reduced by 64.8%, the output is 1.8 times that of the traditional working face, and the advancing speed of the working face is increased by more than 75%.

(4) Compared to the adjacent traditional fully mechanized coal mining face, 16,108 intelligent fully mechanized coal mining faces have great advantages in equipment maintenance, equipment operation mode, and working face efficiency. The successful implementation of this project improves the equipment and technical mining level of thin coal seam, and has a significant and far-reaching impact on the development of thin coal seam mining technology in China.

## Figures and Tables

**Table 1:** 16,108 intelligent fully mechanized mining face and transportation roadway equipment model.

Equipment Name	Equipment Model	Quantity	Whether It Is Automatically Controlled or Not
Shearer	MG400/870-WD double drum type	1	Yes
Hydraulic support	Model ZY4000/09/18D	96	Yes
	Model ZT4000/12/24YD	4	Yes
Scraper conveyor	Model SGZ764/500	1	Yes
Bridge loader	Model SZZ730/160	1	Yes
Crusher	PLM1000	1	Yes
Emulsion pump	Model BRW400/37.5	2	Yes
Spray pump	Type BPW-320/12.5	5	Yes
Water purification equipment	Model JXGSZ-70JB-4A	1	Yes

## Data Availability

No new data were created or analyzed in this study. Data sharing is not applicable to this article.

## References

[1] Sun Q., Geng J., Zhao F. (2020). Experiment Study of Physical and Mechanical Properties of Sandstone After Variable Thermal Cycles. Bull. Eng. Geol. Environ..

[2] Zhang K., Kang L., Chen X., He M., Zhu C., Li D. (2022). A Review of Intelligent Unmanned Mining Current Situation and Development Trend. Energies.

[3] Wo X., Li G., Sun Y., Li J., Yang S., Hao H. (2022). The Changing Tendency and Association Analysis of Intelligent Coal Mines in China: A Policy Text Mining Study. Sustainability.

[4] Tian Z., Zhang Z., Deng M., Yan S., Bai J. (2020). Gob-Side Entry Retained with Soft Roof, Floor, and Seam in Thin Coal Seams: A Case Study. Sustainability.

[5] Wang C., Li H., Zhang M., Liao C., Zhang S. (2023). Characteristics of overlying strata and mechanisms of arch beam failure in shallowly buried thick bedrock coal seams: A case study in western China. Energy Sci. Eng..

[6] Chen W., Jie Z. (2021). New advances in automatic shearer cutting technology for thin seams in Chinese underground coal mines. Energy Explor. Exploit..

[7] Li H., Zu H., Zhang K., Qian J. (2022). Study on Filling Support Design and Ground Pressure Monitoring Scheme for Gob-Side Entry Retention by Roof Cutting and Pressure Relief in High-Gas Thin Coal Seam. Int. J. Environ. Res. Public Health.

[8] Malashkevych D., Petlovanyi M., Sai K., Zubko S. (2022). Research into the coal quality with a new selective mining technology of the waste rock accumulation in the mined-out area. Min. Miner. Deposits.

[9] He L., Yuan D., Ren L., Huang M., Zhang W., Tan J. (2023). Evaluation Model Research of Coal Mine Intelligent Construction Based on Fdematel-Anp. Sustainability.

[10] Li S., Chen J., Liu C. (2022). Overview on the Development of Intelligent Methods for Mineral Resource Prediction under the Background of Geological Big Data. Minerals.

[11] Dong L.J., Wang J., Wang J.C., Wang H.W. (2023). Safe and Intelligent Mining: Some Explorations and Challenges in the Era of Big Data. J. Cent. South Univ..

[12] Li A., Zhang J., Zhou N., Li M., Zhang W. (2020). A Model for Evaluating the Production System of an Intelligent Mine Based on Unascertained Measurement Theory. J. Intell. Fuzzy Syst..

[13] Li J., Zhan K. (2018). Intelligent Mining Technology for an Underground Metal Mine Based on Unmanned Equipment. Engineering.

[14] Petlovanyi V., Malashkevych D.S., Sai K.S. (2020). The new approach to creating progressive and low-waste mining technology for thin coal seams. J. Geol. Geogr. Geoecol..

[15] Bing Z., Wang X., Dong Z., Dong L., He T. (2022). A Novel Edge Computing Architecture for Intelligent Coal Mining System. Wirel. Netw..

[16] Yuan X., Wu Y., Sun L., Wang X. (2023). Research on Efficient Construction Paths for Intelligent Coal Mines in China from the Configuration Perspective. Appl. Sci..

[17] Chen Q., Zou B., Tao Z., He M., Hu B. (2023). Construction and Application of an Intelligent Roof Stability Evaluation System for the Roof-Cutting Non-Pillar Mining Method. Sustainability.

[18] Chen W., Wang X. (2021). Coal Mine Safety Intelligent Monitoring Based on Wireless Sensor Network. IEEE Sens. J..

[19] Yin H., Guo G., Li H., Wu Z. (2022). An Intelligent Optimization Design Method of the Compressed Ratio of Backfilling Body to Avoid Backfilling Mining-Induced Environmental Damage. Environ. Sci. Pollut. Res..

[20] Ma H., Xue W. (2023). Research on the System of Mine Intelligent Positioning. Wirel. Pers. Commun..

[21] Wu C.A., Lin W.Y., Jiang C.L., Wu C.C. (2011). Toward Intelligent Data Warehouse Mining: An Ontology-Integrated Approach for Multi-dimensional Association Mining. Expert Syst. Appl..

[22] Wang J., Huang Z. (2017). The Recent Technological Development of Intelligent Mining in China. Engineering.

[23] Hakan B., Fend O.I., Osman A. (2015). Prediction of the Stresses Around Main and Tail Gates During Top Coal Caving by 3D Numerical Analysis. Int. J. Rock Mech. Min. Sci..

[24] Massinaei M., Jahedsaravani A., Taheri E., Khalilpour J. (2018). Machine vision based monitoring and analysis of a coal column flotation circuit. Powder Technol..

[25] Tian Y., Yang X., Yang J., Mao K., Yao Y., Liang H. (2022). Evolution Dynamic of Intellgent Construction Strategy of Coal Mine Enterprises in China. Heliyon.

[26] Shen Y., Li Y., Li Z. (2022). Application of Intelligent Inspection Robot in Coal Mine Industrial Heritage Landscape: Taking Wangshiwa Coal Mine as an Example. Front. Neurorobot..

[27] Yang Y., Zeng Q., Zhang Q. (2023). Analysis of Coal Gangue Recognition Capability Based on Vibration Characteristics of the Tail Beam and Experimental Study on Coal Gangue Recognition in Fully Mechanized Top Coal Caving. Int. J. Coal Prep. Util..

[28] Zadkov D., Gabov V., Babyr N., Stebnev A., Teremetskaya V. (2022). Adaptable and energy-efficient powered roof support unit. Min. Inf. Anal. Bull..

[29] Wu X., Li H., Wang B., Zhu M. (2022). Review on Improvements to the Safety Level of Coal Mines by Applying Intelligent Coal Mining. Sustainability.

[30] Huang X., Liu Y. (2023). Research and Design of Intelligent Mine Ventilation Construction Architecture. Int. J. Low-Carbon Technol..

[31] Zhan P. (2023). Application of 5G Communication Technology Based on Intelligent Sensor Network in Coal Mining. J. Sens..

[32] Si L., Xiong X., Wang Z., Tan C. (2020). A Deep Convolutional Neural Network Model for Intelligent Discrimination between Coal and Rocks in Coal Mining Face. Math. Probl. Eng..

[33] Bołoz Ł. (2018). Mining of thin coal seams using surface-underground methods. Min. Inform. Autom. Electr. Eng..

[34] Babyr N., Babyr K. (2021). To improve the contact adaptability of mechanical roof support. E3S Web of Conferences.

